# Crystal structure of (ferrocenylmeth­yl)di­methyl­ammonium hydrogen oxalate

**DOI:** 10.1107/S205698901501333X

**Published:** 2015-07-17

**Authors:** Mamadou Ndiaye, Abdoulaye Samb, Libasse Diop, Thierry Maris

**Affiliations:** a Laboratoire des Produits Naturels, Département de Chimie, Faculté des Sciences et Techniques, Université Cheikh Anta Diop, Dakar, Sénégal; b Laboratoire de Chimie Minérale et Analytique, Département de Chimie, Faculté des Sciences et Techniques, Université Cheikh Anta Diop, Dakar, Sénégal; c Département de Chimie, Université de Montréal, 2900 Boulevard Édouard-Montpetit, Montréal, Québec, H3C 3J7, Canada

**Keywords:** crystal structure, hydrogen oxalate, (ferrocenylmeth­yl)di­methyl­ammonium, bifurcated hydrogen bond, C—H⋯π inter­actions

## Abstract

The crystal structure of the title salt, [Fe(C_5_H_5_)(C_8_H_13_N)](HC_2_O_4_), consists of discrete (ferrocenylmeth­yl)di­methyl­ammonium cations and hydrogen oxalate anions. The anions are connected through a strong O—H⋯O hydrogen bond, forming linear chains running parallel to [100]. The cations are linked to the anions through bifurcated N—H⋯(O,O′) hydrogen bonds. Weak C—H⋯π inter­actions between neighbouring ferrocenyl moieties are also observed.

## Abstract 2

In the title salt, [Fe(C_5_H_5_)(C_8_H_13_N)](HC_2_O_4_), the anions are linked *via* strong O—H⋯O hydrogen bonds into linear [100] chains. The cations connect to the anion through bifurcated N—H⋯(O,O′) hydrogen bonds.

## Chemical context   

Our group has been working on the inter­actions between alkyl­ammonium ions with oxalic acid, and we have recently reported the crystal structure of (H_3_C)_2_NH^+^·HC_2_O_4_^−^·0.5H_2_C_2_O_4_ (Diallo *et al.*, 2015 ▸). Numerous other reports have described crystal structures containing acidic or neutral oxalate mol­ecules inter­acting with a protonated amine, see for example: Vaidhyanathan *et al.* (2002 ▸); Braga *et al.* (2013 ▸); Said *et al.* (2006 ▸); Hathwar *et al.* (2010 ▸); Matulková *et al.* (2008 ▸); Olenik *et al.* (2003 ▸); Anda *et al.* (2004 ▸). Braga *et al.* have reported several structures of columnar metallocenium sandwich compounds inter­acting with hydrogen oxalate (Braga *et al.*, 2002 ▸). However, none of these structures features the hydrogen oxalate anion alone. It is crystallized either with neutral oxalic acid and/or a water mol­ecule. The crystal structure of the title salt, [Fe(C_5_H_5_)(C_8_H_13_N)]^+^·[HC_2_O_4_]^−^, (I), features only the hydrogen oxalate anion. This compound was obtained when studying the inter­action of (ferrocenylmeth­yl)di­methyl­amine and oxalic acid in aqueous solution.
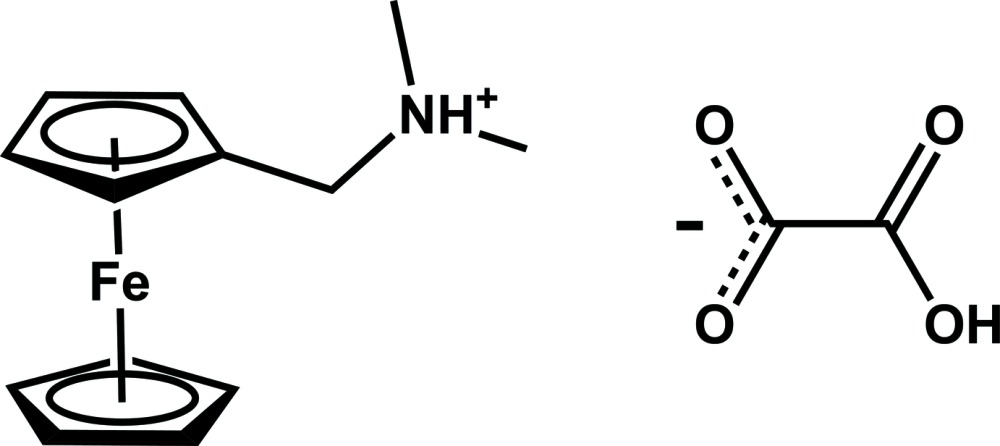


## Structural commentary   

**Figure 1:**
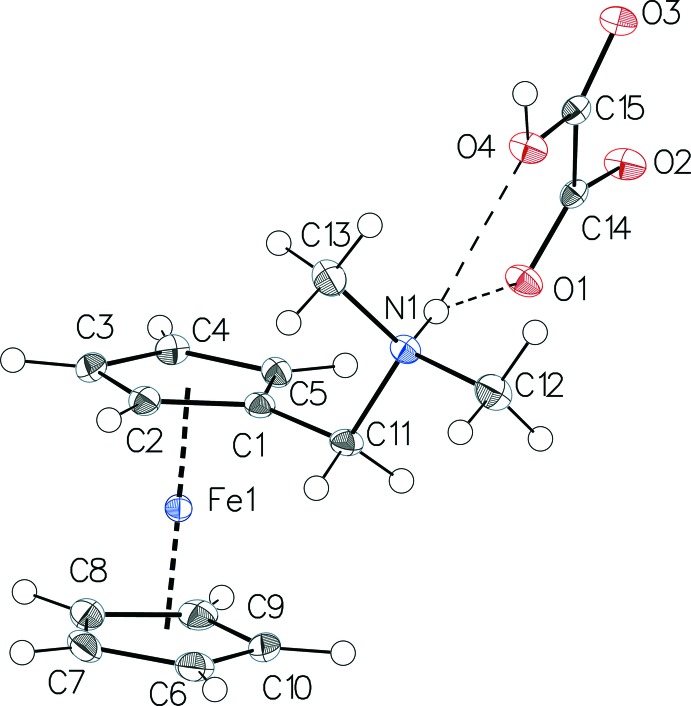
The mol­ecular components in the structure of the title compound, with displacement ellipsoids drawn at the 50% probability level. H atoms are shown as small spheres of arbitrary radii. Fe–Cp inter­actions and hydrogen bonds are shown as dashed lines.

The asymmetric unit of (I) contains one hydrogen oxalate anion and one (ferrocenylmeth­yl)di­methyl­ammonium cation (Fig. 1 ▸). As previously observed in structures featuring this cation (Wang, 2010 ▸; Guo, 2006 ▸; Guo *et al.*, 2006*a* ▸,*b* ▸), the two Cp rings exhibit a nearly eclipsed conformation. They are planar and almost parallel, as demonstrated by the dihedral angle of 0.96 (5)° between their least-square planes. The Fe—C distances range from 2.0394 (10) to 2.0578 (12) Å. The Fe binding with the Cp rings is somewhat asymmetric as suggested by both the Fe⋯Cp plane distances [1.6601 (6) and 1.6514 (6) Å for the unsubstituted and the substituted ligand, respectively], and the Cp1–Fe–Cp2 dihedral angle of 170.96 (3)°. This behaviour was previously described as a consequence of an electron-withdrawal effect of the methyl­dimethyl­amine group that results in the less electron-rich substituted ring being slightly closer to the metal (Winter & Wolmershäuser, 1998 ▸). The oxalate anion is essentially planar and the dihedral angle between carboxyl­ate and the carboxyl groups is only 4.6 (3)°. The C—OH bond is at 1.3052 (13) significantly longer than the other three C—O bonds with an mean of 1.24 (2) Å.

## Supra­molecular features   

**Figure 2:**
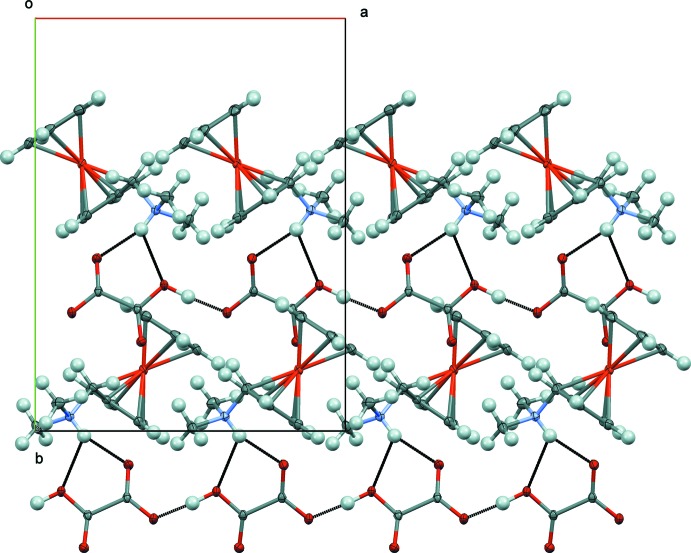
Partial packing diagram in the structure of the title compound viewed along [001]. The chains running along [100] as defined by the hydrogen-bonded hydrogen oxalate anions and the (ferrocenylmeth­yl)di­methyl­ammonium cations linked by a bifurcated hydrogen bond are shown. Hydrogen bonds are shown as black lines.

The hydrogen oxalate anions are held together *via* a strong inter­molecular O4—H4*A*⋯O2 hydrogen bond, resulting in the formation of linear chains running parallel to [100] (Fig. 2 ▸). Within a chain, successive hydrogen oxalate anions are rotated by 30.89 (11)°. The cation is linked to the anionic chain through a bifurcated N1—H1⋯(O1,O4) hydrogen bond (Table 1 ▸). In addition to Coulomb forces and hydrogen bonds, a weak C—H⋯π inter­action involving the centroid *Cg*2 of the Cp ligand (C6–C10; Table 1 ▸) is present and consolidates the three-dimensional supra­molecular network.

## Database survey   

A search in the Cambridge Structural database (Version 5.36 with three updates, Groom & Allen, 2014 ▸) returned only eight entries for seven independent crystal structures containing the (ferrocenylmeth­yl)di­methyl­ammonium cation. These include simple salts with Cl^−^ (Winter & Wolmershäuser, 1998 ▸) and its hydrated form (Guo *et al.*, 2006*a* ▸), Br^−^ (Wang, 2010 ▸), NO_3_^−^ (Guo *et al.*, 2006*b* ▸), sulfate penta­hydrate (Guo, 2006 ▸), tetra­chlorido­zincate monohydrate (Gibbons & Trotter, 1971 ▸) and a benzene solvate with dodeca­borane (Yongmao *et al.*, 1983 ▸). The investigation of hydrogen-bonded hydrogen oxalate chains returned 119 unique structures of which 32 are characterized by a bifurcated hydrogen bond with an ammonium counter-cation.

## Synthesis and crystallization   

Crystals of the title compound were obtained by slow evaporation of an aqueous solution in which (ferrocenylmeth­yl)di­methyl­amine was mixed with oxalic acid in a 1:2 ratio.

## Refinement   

Crystal data, data collection and structure refinement details are summarized in Table 2 ▸. All hydrogen atoms were located in difference Fourier maps and were freely refined.

## Supplementary Material

Crystal structure: contains datablock(s) I. DOI: 10.1107/S205698901501333X/wm5182sup1.cif

Structure factors: contains datablock(s) I. DOI: 10.1107/S205698901501333X/wm5182Isup2.hkl

CCDC reference: 1412152

Additional supporting information:  crystallographic information; 3D view; checkCIF report

## Figures and Tables

**Table 1:** Hydrogen-bond geometry (, ) *Cg*2 is the centroid of the Cp ligand C6C10.

*D*H*A*	*D*H	H*A*	*D* *A*	*D*H*A*
N1H1O1	0.878(15)	1.981(15)	2.8180(11)	158.9(14)
N1H1O4	0.878(15)	2.346(15)	2.8958(11)	120.8(11)
O4H4*A*O2^i^	0.96(2)	1.52(2)	2.4776(11)	174.1(18)
C2H2*Cg*2^ii^	0.935(19)	2.743(19)	3.6564(13)	165.8(13)

Symmetry codes: (i) 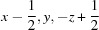; (ii) 
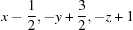
.

**Table 2:** Experimental details

Crystal data	
Chemical formula	[Fe(C_5_H_5_)(C_8_H_13_N)](C_2_HO_4_)
*M*_r_	333.16
Crystal system, space group	Orthorhombic, *P* *b* *c* *a*
Temperature (K)	100
*a*, *b*, *c* ()	11.2225(3), 14.8991(4), 17.2727(5)
*V* (^3^)	2888.08(14)
*Z*	8
Radiation type	Ga *K*, = 1.34139
(mm^1^)	5.72
Crystal size (mm)	0.15 0.13 0.09

Data collection	
Diffractometer	Bruker Venture Metaljet
Absorption correction	Multi-scan (*SADABS*; Krause *et al.*, 2015 ▸)
*T*_min_, *T*_max_	0.585, 0.752
No. of measured, independent and observed [*I* > 2(*I*)] reflections	56674, 3323, 3150
*R*_int_	0.034
(sin /)_max_ (^1^)	0.650

Refinement	
*R*[*F* ^2^ > 2(*F* ^2^)], *wR*(*F* ^2^), *S*	0.022, 0.062, 1.03
No. of reflections	3323
No. of parameters	266
H-atom treatment	All H-atom parameters refined
_max_, _min_ (e ^3^)	0.45, 0.19

Computer programs: *APEX2* and *SAINT* (Bruker, 2014 ▸), *SHELXT* (Sheldrick, 2015*a* ▸), *SHELXL2014* (Sheldrick, 2015*b* ▸), *OLEX2* (Dolomanov *et al.*, 2009 ▸), *Mercury* (Macrae *et al.*, 2008 ▸) and *publCIF* (Westrip, 2010 ▸).

## supplementary crystallographic information

### Crystal data

**Table:**

[Fe(C_5_H_5_)(C_8_H_13_N)](C_2_HO_4_)	*D*_x_ = 1.532 Mg m^−^^3^
*M**_r_* = 333.16	Ga *K*α radiation, λ = 1.34139 Å
Orthorhombic, *P**b**c**a*	Cell parameters from 9948 reflections
*a* = 11.2225 (3) Å	θ = 4.8–60.7°
*b* = 14.8991 (4) Å	µ = 5.72 mm^−^^1^
*c* = 17.2727 (5) Å	*T* = 100 K
*V* = 2888.08 (14) Å^3^	Block, clear light orange
*Z* = 8	0.14 × 0.13 × 0.09 mm
*F*(000) = 1392	

### Data collection

**Table:**

Bruker Venture Metaljet diffractometer	3323 independent reflections
Radiation source: Metal Jet, Gallium Liquid Metal Jet Source	3150 reflections with *I* > 2σ(*I*)
Helios MX Mirror Optics monochromator	*R*_int_ = 0.034
Detector resolution: 10.24 pixels mm^-1^	θ_max_ = 60.7°, θ_min_ = 4.5°
ω and φ scans	*h* = −14→14
Absorption correction: multi-scan (*SADABS*; Krause *et al.*, 2015)	*k* = −17→19
*T*_min_ = 0.585, *T*_max_ = 0.752	*l* = −22→22
56674 measured reflections	

### Refinement

**Table:**

Refinement on *F*^2^	Primary atom site location: structure-invariant direct methods
Least-squares matrix: full	Hydrogen site location: difference Fourier map
*R*[*F*^2^ > 2σ(*F*^2^)] = 0.022	All H-atom parameters refined
*wR*(*F*^2^) = 0.062	*w* = 1/[σ^2^(*F*_o_^2^) + (0.0388*P*)^2^ + 0.8916*P*] where *P* = (*F*_o_^2^ + 2*F*_c_^2^)/3
*S* = 1.03	(Δ/σ)_max_ = 0.002
3323 reflections	Δρ_max_ = 0.45 e Å^−^^3^
266 parameters	Δρ_min_ = −0.19 e Å^−^^3^
0 restraints	

### Special details

**Table:**

Experimental. X-ray crystallographic data for I were collected from a single-crystal sample, which was mounted on a loop fiber. Data were collected using a Bruker Venture diffractometer equipped with a Photon 100 CMOS Detector, a Helios MX optics and a Kappa goniometer. The crystal-to-detector distance was 4.0 cm, and the data collection was carried out in 1024 *x* 1024 pixel mode.
Geometry. All e.s.d.'s (except the e.s.d. in the dihedral angle between two l.s. planes) are estimated using the full covariance matrix. The cell e.s.d.'s are taken into account individually in the estimation of e.s.d.'s in distances, angles and torsion angles; correlations between e.s.d.'s in cell parameters are only used when they are defined by crystal symmetry. An approximate (isotropic) treatment of cell e.s.d.'s is used for estimating e.s.d.'s involving l.s. planes.

### Fractional atomic coordinates and isotropic or equivalent isotropic displacement parameters (Å^2^)

**Table:**

	*x*	*y*	*z*	*U*_iso_*/*U*_eq_	
Fe1	0.34931 (2)	0.64806 (2)	0.49469 (2)	0.01187 (7)	
N1	0.11135 (8)	0.53481 (6)	0.31389 (5)	0.01286 (17)	
C1	0.22887 (9)	0.57630 (6)	0.43156 (6)	0.01299 (19)	
C2	0.17883 (11)	0.60032 (8)	0.50499 (6)	0.0150 (2)	
C3	0.25259 (10)	0.56311 (7)	0.56398 (6)	0.0164 (2)	
C4	0.34734 (9)	0.51541 (7)	0.52776 (7)	0.0161 (2)	
C5	0.33275 (9)	0.52304 (7)	0.44578 (6)	0.0143 (2)	
C6	0.35605 (10)	0.77834 (8)	0.45550 (7)	0.0199 (2)	
C7	0.35680 (10)	0.77649 (8)	0.53827 (7)	0.0199 (2)	
C8	0.45806 (10)	0.72650 (7)	0.56226 (6)	0.0193 (2)	
C9	0.52010 (11)	0.69741 (8)	0.49491 (6)	0.0193 (2)	
C10	0.45688 (11)	0.72947 (7)	0.42883 (6)	0.0200 (2)	
C11	0.18587 (9)	0.60523 (7)	0.35384 (6)	0.0148 (2)	
C12	0.07459 (11)	0.56695 (8)	0.23563 (7)	0.0209 (2)	
C13	0.00554 (10)	0.50895 (8)	0.36082 (7)	0.0218 (2)	
O1	0.30103 (6)	0.41777 (5)	0.27870 (5)	0.01774 (16)	
O2	0.38177 (7)	0.28665 (5)	0.24336 (5)	0.01896 (17)	
O3	0.15744 (6)	0.21661 (5)	0.23000 (5)	0.01636 (16)	
O4	0.08414 (7)	0.35313 (5)	0.25702 (5)	0.01652 (16)	
C14	0.29562 (9)	0.33826 (7)	0.25759 (6)	0.01189 (19)	
C15	0.16996 (9)	0.29528 (7)	0.24647 (6)	0.01225 (19)	
H11A	0.1366 (12)	0.6592 (9)	0.3575 (8)	0.015 (3)*	
H5	0.3823 (13)	0.4963 (9)	0.4076 (8)	0.019 (3)*	
H9	0.5900 (18)	0.6637 (12)	0.4949 (8)	0.029 (4)*	
H4	0.4109 (12)	0.4842 (9)	0.5532 (8)	0.017 (3)*	
H6	0.2957 (14)	0.8082 (10)	0.4249 (9)	0.025 (4)*	
H1	0.1581 (12)	0.4881 (10)	0.3079 (9)	0.024 (4)*	
H2	0.1122 (18)	0.6365 (12)	0.5139 (9)	0.030 (4)*	
H11B	0.2507 (12)	0.6154 (9)	0.3202 (8)	0.017 (3)*	
H8	0.4786 (14)	0.7118 (10)	0.6155 (9)	0.029 (4)*	
H12A	0.0229 (15)	0.6170 (11)	0.2419 (9)	0.032 (4)*	
H3	0.2436 (14)	0.5700 (9)	0.6199 (9)	0.027 (4)*	
H13A	−0.0415 (15)	0.4691 (11)	0.3319 (9)	0.034 (4)*	
H10	0.4764 (15)	0.7189 (10)	0.3752 (9)	0.032 (4)*	
H13B	0.0345 (15)	0.4801 (11)	0.4091 (10)	0.033 (4)*	
H7	0.2965 (14)	0.8042 (10)	0.5702 (9)	0.028 (4)*	
H13C	−0.0372 (14)	0.5622 (10)	0.3732 (9)	0.029 (4)*	
H12B	0.1486 (14)	0.5830 (11)	0.2080 (10)	0.031 (4)*	
H12C	0.0350 (13)	0.5191 (10)	0.2088 (8)	0.021 (3)*	
H4A	0.007 (2)	0.3250 (12)	0.2542 (11)	0.049 (5)*	

### Atomic displacement parameters (Å^2^)

**Table:**

	*U*^11^	*U*^22^	*U*^33^	*U*^12^	*U*^13^	*U*^23^
Fe1	0.01085 (10)	0.01291 (10)	0.01184 (10)	−0.00209 (5)	0.00031 (5)	−0.00109 (5)
N1	0.0108 (4)	0.0125 (4)	0.0152 (4)	−0.0001 (3)	−0.0009 (3)	−0.0013 (3)
C1	0.0113 (4)	0.0122 (4)	0.0155 (5)	−0.0030 (4)	0.0000 (4)	−0.0007 (4)
C2	0.0121 (5)	0.0148 (5)	0.0182 (5)	−0.0015 (4)	0.0028 (4)	−0.0012 (4)
C3	0.0174 (5)	0.0168 (5)	0.0150 (5)	−0.0041 (4)	0.0022 (4)	0.0004 (4)
C4	0.0156 (5)	0.0150 (5)	0.0175 (5)	−0.0010 (4)	−0.0016 (4)	0.0016 (4)
C5	0.0122 (4)	0.0145 (5)	0.0160 (5)	−0.0008 (4)	0.0006 (4)	−0.0025 (4)
C6	0.0229 (6)	0.0147 (5)	0.0220 (6)	−0.0039 (4)	−0.0035 (4)	0.0015 (4)
C7	0.0211 (5)	0.0164 (5)	0.0222 (6)	−0.0040 (4)	0.0007 (4)	−0.0064 (4)
C8	0.0200 (5)	0.0206 (5)	0.0173 (5)	−0.0078 (4)	−0.0029 (4)	−0.0012 (4)
C9	0.0131 (5)	0.0184 (5)	0.0264 (6)	−0.0052 (4)	0.0008 (4)	−0.0013 (4)
C10	0.0238 (6)	0.0191 (5)	0.0170 (5)	−0.0093 (4)	0.0039 (4)	−0.0012 (4)
C11	0.0151 (5)	0.0127 (5)	0.0167 (5)	−0.0034 (4)	−0.0027 (4)	0.0002 (4)
C12	0.0258 (6)	0.0178 (5)	0.0189 (5)	0.0016 (4)	−0.0083 (5)	0.0000 (4)
C13	0.0142 (5)	0.0258 (6)	0.0254 (6)	−0.0067 (5)	0.0060 (4)	−0.0091 (5)
O1	0.0121 (3)	0.0154 (3)	0.0257 (4)	−0.0010 (3)	0.0006 (3)	−0.0054 (3)
O2	0.0099 (3)	0.0153 (4)	0.0317 (4)	0.0007 (3)	0.0018 (3)	−0.0011 (3)
O3	0.0143 (4)	0.0129 (3)	0.0219 (4)	−0.0006 (3)	−0.0025 (3)	−0.0002 (3)
O4	0.0088 (3)	0.0149 (4)	0.0258 (4)	0.0005 (3)	−0.0001 (3)	−0.0020 (3)
C14	0.0099 (4)	0.0146 (4)	0.0112 (4)	−0.0006 (4)	−0.0001 (3)	0.0024 (4)
C15	0.0106 (4)	0.0142 (4)	0.0120 (4)	−0.0001 (4)	−0.0008 (3)	0.0022 (4)

### Geometric parameters (Å, º)

**Table:**

Fe1—C1	2.0394 (10)	C6—C7	1.4298 (19)
Fe1—C2	2.0490 (12)	C6—C10	1.4223 (17)
Fe1—C3	2.0524 (10)	C6—H6	0.967 (16)
Fe1—C4	2.0574 (11)	C7—C8	1.4206 (17)
Fe1—C5	2.0538 (11)	C7—H7	0.965 (16)
Fe1—C6	2.0570 (12)	C8—C9	1.4233 (16)
Fe1—C7	2.0578 (12)	C8—H8	0.974 (16)
Fe1—C8	2.0536 (11)	C9—C10	1.4263 (16)
Fe1—C9	2.0528 (12)	C9—H9	0.93 (2)
Fe1—C10	2.0549 (11)	C10—H10	0.965 (16)
N1—C11	1.5087 (12)	C11—H11A	0.978 (13)
N1—C12	1.4923 (13)	C11—H11B	0.943 (14)
N1—C13	1.4885 (13)	C12—H12A	0.951 (17)
N1—H1	0.878 (15)	C12—H12B	0.987 (17)
C1—C2	1.4324 (14)	C12—H12C	0.959 (15)
C1—C5	1.4316 (14)	C13—H13A	0.939 (17)
C1—C11	1.4902 (14)	C13—H13B	0.992 (17)
C2—C3	1.4251 (15)	C13—H13C	0.951 (15)
C2—H2	0.935 (19)	O1—C14	1.2410 (12)
C3—C4	1.4237 (15)	O2—C14	1.2595 (13)
C3—H3	0.976 (15)	O3—C15	1.2144 (14)
C4—C5	1.4299 (15)	O4—C15	1.3052 (13)
C4—H4	0.958 (14)	O4—H4A	0.96 (2)
C5—H5	0.950 (15)	C14—C15	1.5607 (14)
			
C1—Fe1—C2	41.02 (4)	C4—C3—H3	124.4 (9)
C1—Fe1—C3	68.77 (4)	Fe1—C4—H4	125.8 (8)
C1—Fe1—C4	68.76 (4)	C3—C4—Fe1	69.54 (6)
C1—Fe1—C5	40.94 (4)	C3—C4—C5	108.05 (9)
C1—Fe1—C6	110.06 (4)	C3—C4—H4	126.7 (8)
C1—Fe1—C7	135.24 (4)	C5—C4—Fe1	69.51 (6)
C1—Fe1—C8	174.93 (4)	C5—C4—H4	125.3 (8)
C1—Fe1—C9	143.86 (4)	Fe1—C5—H5	127.8 (8)
C1—Fe1—C10	113.75 (4)	C1—C5—Fe1	68.99 (6)
C2—Fe1—C3	40.66 (4)	C1—C5—H5	126.2 (9)
C2—Fe1—C4	68.43 (4)	C4—C5—Fe1	69.78 (6)
C2—Fe1—C5	68.67 (4)	C4—C5—C1	107.89 (9)
C2—Fe1—C6	112.98 (5)	C4—C5—H5	125.9 (9)
C2—Fe1—C7	109.22 (4)	Fe1—C6—H6	126.0 (9)
C2—Fe1—C8	134.68 (4)	C7—C6—Fe1	69.70 (6)
C2—Fe1—C9	174.88 (4)	C7—C6—H6	124.0 (9)
C2—Fe1—C10	143.28 (5)	C10—C6—Fe1	69.68 (6)
C3—Fe1—C4	40.54 (4)	C10—C6—C7	108.02 (10)
C3—Fe1—C5	68.45 (4)	C10—C6—H6	128.0 (9)
C3—Fe1—C6	142.49 (5)	Fe1—C7—H7	125.3 (9)
C3—Fe1—C7	112.44 (5)	C6—C7—Fe1	69.64 (6)
C3—Fe1—C8	109.50 (4)	C6—C7—H7	124.0 (9)
C3—Fe1—C9	135.53 (5)	C8—C7—Fe1	69.63 (6)
C3—Fe1—C10	175.95 (5)	C8—C7—C6	107.85 (10)
C4—Fe1—C7	142.38 (5)	C8—C7—H7	128.2 (9)
C5—Fe1—C4	40.71 (4)	Fe1—C8—H8	123.4 (9)
C5—Fe1—C6	136.37 (5)	C7—C8—Fe1	69.95 (6)
C5—Fe1—C7	175.92 (5)	C7—C8—C9	108.21 (10)
C5—Fe1—C10	111.15 (4)	C7—C8—H8	125.7 (9)
C6—Fe1—C4	176.56 (5)	C9—C8—Fe1	69.69 (6)
C6—Fe1—C7	40.67 (5)	C9—C8—H8	126.0 (9)
C8—Fe1—C4	113.28 (5)	Fe1—C9—H9	126.3 (11)
C8—Fe1—C5	143.48 (5)	C8—C9—Fe1	69.75 (6)
C8—Fe1—C6	68.18 (5)	C8—C9—C10	107.99 (11)
C8—Fe1—C7	40.43 (5)	C8—C9—H9	125.2 (9)
C8—Fe1—C10	68.26 (5)	C10—C9—Fe1	69.76 (6)
C9—Fe1—C4	110.71 (5)	C10—C9—H9	126.8 (9)
C9—Fe1—C5	114.21 (4)	Fe1—C10—H10	124.6 (9)
C9—Fe1—C6	68.18 (5)	C6—C10—Fe1	69.84 (6)
C9—Fe1—C7	68.17 (5)	C6—C10—C9	107.94 (10)
C9—Fe1—C8	40.56 (4)	C6—C10—H10	125.1 (10)
C9—Fe1—C10	40.64 (5)	C9—C10—Fe1	69.60 (6)
C10—Fe1—C4	136.64 (5)	C9—C10—H10	126.9 (10)
C10—Fe1—C6	40.47 (5)	N1—C11—H11A	106.7 (8)
C10—Fe1—C7	68.26 (5)	N1—C11—H11B	104.9 (8)
C11—N1—H1	105.9 (9)	C1—C11—N1	112.98 (8)
C12—N1—C11	110.15 (8)	C1—C11—H11A	111.3 (9)
C12—N1—H1	108.2 (10)	C1—C11—H11B	110.6 (8)
C13—N1—C11	111.91 (8)	H11A—C11—H11B	110.1 (11)
C13—N1—C12	110.84 (9)	N1—C12—H12A	108.5 (9)
C13—N1—H1	109.6 (10)	N1—C12—H12B	106.5 (10)
C2—C1—Fe1	69.85 (6)	N1—C12—H12C	109.1 (8)
C2—C1—C11	126.76 (9)	H12A—C12—H12B	112.3 (14)
C5—C1—Fe1	70.07 (6)	H12A—C12—H12C	110.8 (13)
C5—C1—C2	107.80 (9)	H12B—C12—H12C	109.7 (13)
C5—C1—C11	125.35 (9)	N1—C13—H13A	108.8 (10)
C11—C1—Fe1	123.00 (7)	N1—C13—H13B	107.9 (10)
Fe1—C2—H2	124.2 (11)	N1—C13—H13C	108.0 (9)
C1—C2—Fe1	69.13 (6)	H13A—C13—H13B	111.0 (13)
C1—C2—H2	127.2 (10)	H13A—C13—H13C	111.3 (13)
C3—C2—Fe1	69.80 (6)	H13B—C13—H13C	109.7 (12)
C3—C2—C1	107.95 (10)	C15—O4—H4A	111.6 (11)
C3—C2—H2	124.8 (10)	O1—C14—O2	127.06 (10)
Fe1—C3—H3	124.5 (9)	O1—C14—C15	118.16 (9)
C2—C3—Fe1	69.54 (6)	O2—C14—C15	114.78 (8)
C2—C3—H3	127.3 (9)	O3—C15—O4	125.78 (9)
C4—C3—Fe1	69.92 (6)	O3—C15—C14	121.97 (9)
C4—C3—C2	108.29 (10)	O4—C15—C14	112.25 (8)
			
Fe1—C1—C2—C3	−59.20 (8)	C5—C1—C11—N1	83.77 (12)
Fe1—C1—C5—C4	59.14 (7)	C6—C7—C8—Fe1	−59.36 (7)
Fe1—C1—C11—N1	171.61 (7)	C6—C7—C8—C9	0.04 (12)
Fe1—C2—C3—C4	−59.38 (7)	C7—C6—C10—Fe1	59.37 (7)
Fe1—C3—C4—C5	−59.03 (7)	C7—C6—C10—C9	−0.03 (12)
Fe1—C4—C5—C1	−58.64 (7)	C7—C8—C9—Fe1	−59.56 (8)
Fe1—C6—C7—C8	59.36 (7)	C7—C8—C9—C10	−0.06 (13)
Fe1—C6—C10—C9	−59.40 (8)	C8—C9—C10—Fe1	−59.50 (8)
Fe1—C7—C8—C9	59.40 (8)	C8—C9—C10—C6	0.05 (13)
Fe1—C8—C9—C10	59.50 (8)	C10—C6—C7—Fe1	−59.36 (7)
Fe1—C9—C10—C6	59.55 (8)	C10—C6—C7—C8	0.00 (11)
C1—C2—C3—Fe1	58.79 (8)	C11—C1—C2—Fe1	−116.75 (10)
C1—C2—C3—C4	−0.59 (13)	C11—C1—C2—C3	−175.95 (9)
C2—C1—C5—Fe1	−59.91 (7)	C11—C1—C5—Fe1	116.95 (10)
C2—C1—C5—C4	−0.78 (11)	C11—C1—C5—C4	176.08 (9)
C2—C1—C11—N1	−99.97 (12)	C12—N1—C11—C1	−178.23 (9)
C2—C3—C4—Fe1	59.15 (8)	C13—N1—C11—C1	57.99 (12)
C2—C3—C4—C5	0.11 (12)	O1—C14—C15—O3	−175.95 (10)
C3—C4—C5—Fe1	59.05 (7)	O1—C14—C15—O4	3.94 (13)
C3—C4—C5—C1	0.41 (11)	O2—C14—C15—O3	4.30 (14)
C5—C1—C2—Fe1	60.05 (7)	O2—C14—C15—O4	−175.81 (9)
C5—C1—C2—C3	0.85 (12)		

### Hydrogen-bond geometry (Å, º)

Cg2 is the centroid of the Cp ligand C6–C10.

**Table:**

*D*—H···*A*	*D*—H	H···*A*	*D*···*A*	*D*—H···*A*
N1—H1···O1	0.878 (15)	1.981 (15)	2.8180 (11)	158.9 (14)
N1—H1···O4	0.878 (15)	2.346 (15)	2.8958 (11)	120.8 (11)
C11—H11*B*···O3^i^	0.943 (14)	2.401 (14)	3.2282 (13)	146.3 (11)
C12—H12*A*···O3^ii^	0.951 (17)	2.556 (17)	3.4792 (14)	163.8 (13)
C13—H13*A*···O4	0.939 (17)	2.578 (16)	3.0631 (14)	112.5 (12)
O4—H4*A*···O2^iii^	0.96 (2)	1.52 (2)	2.4776 (11)	174.1 (18)
C2—H2···*Cg*2^iv^	0.935 (19)	2.743 (19)	3.6564 (13)	165.8 (13)

Symmetry codes: (i) −*x*+1/2, *y*+1/2, *z*; (ii) −*x*, *y*+1/2, −*z*+1/2; (iii) *x*−1/2, *y*, −*z*+1/2; (iv) *x*−1/2, −*y*+3/2, −*z*+1.
